# Evaluation of a differentiation model of preschoolers’ executive functions

**DOI:** 10.3389/fpsyg.2015.00285

**Published:** 2015-03-17

**Authors:** Steven J. Howard, Anthony D. Okely, Yvonne G. Ellis

**Affiliations:** ^1^ School of Education, University of Wollongong, Wollongong, NSWAustralia; ^2^ Early Start Research Institute, University of Wollongong, Wollongong, NSWAustralia

**Keywords:** executive function, preschool, inhibition, working memory, shifting, cognitive development

## Abstract

Despite the prominent role of executive functions in children’s emerging competencies, there remains debate regarding the structure and development of executive functions. In an attempt to reconcile these discrepancies, a differentiation model of executive function development was evaluated in the early years using 6-month age groupings. Specifically, 281 preschoolers completed measures of working memory, inhibition, and shifting. Results contradicted suggestions that executive functions follow a single trajectory of progressive separation in childhood, instead suggesting that these functions may undergo a period of integration in the preschool years. These results highlight potential problems with current practices and theorizing in executive function research.

## Introduction

Integral to a child’s cognitive development is an increasing ability to activate, coordinate, and manipulate information in mind. Current formulations suggest that this is made possible by the capacity and control of attention, which are supported by an executive control network (consisting of the dorsolateral prefrontal cortex, anterior cingulate, supplementary motor area, and basal ganglia; Rothbart and Posner, 2001). In this context, the capacity of attention regards the maximum amount of information that concurrently can be activated within the focus of attention [often termed working memory (WM) capacity; Shipstead et al., 2012]. In contrast, control processes direct the focus of attention toward task-relevant information (e.g., shifting) and away from task-irrelevant information (e.g., inhibition; Shipstead et al., 2012). These processes are routinely bundled as executive functions (Diamond, 2006), given their role in enabling, constraining, directing, and supporting complex cognition. Although the exact course of executive function development remains debated, research suggests that they do not reach maturity until adolescence (Best et al., 2009).

The development of executive functions appears to play a prominent role in children’s emerging academic, social, emotional, and behavioral competencies. For instance, research indicates that children’s executive functioning is related to preparedness for school (Blair and Razza, 2007; Welsh et al., 2010). Academically, superior executive functioning is associated with an early literacy and numeracy advantage (Blair and Razza, 2007), which longitudinal evidence indicates is maintained through at least the first 3 years of schooling (Bull et al., 2008). In fact, early executive functioning predicts learning more generally, across a range of domains (Bull et al., 2008). Individual differences in executive functions also predict children’s social and emotional development (e.g., social understanding, moral conduct; Riggs et al., 2006). The critical role of executive functions in development is further apparent in evidence that deficient executive functioning is often found in a range of developmental disorders (e.g., ADHD; Willcutt et al., 2005). Research thus highlights broad implications of children’s executive function development, which appears to set the stage for a wide range of later developments.

The preschool years have been identified as a particularly crucial time in the emergence and development of executive functions. The preschool period sees increases in WM capacity (Gathercole et al., 2004), as well as duration, and frequency of attentional focus (Kannass et al., 2006). Attentional control also displays rapid development in these formative years, with significant increases in the ability to resist distraction and overcome task-irrelevant pre-potent responding (Carlson, 2005; Diamond, 2006) and a rapid increase in the ability to exercise control over shifts in attention (Carlson, 2005; Zelazo, 2006). In fact, it has been suggested that development of executive functions in the preschool years may reflect a more qualitative change in cognitive function, whereas later developments reflect quantitative refinements and enhancements of these abilities (Best and Miller, 2010).

Despite the integral role that executive functions play in normal and atypical development, there remains debate regarding the development and dissociation of these functions. On the one hand, the fractionated nature of executive functions is supported by the extraction of multiple executive function factors using latent variable approaches (although the quantity, composition, interpretation, and development of these factors is debated; Miyake et al., 2000; Klenberg et al., 2001; Lehto et al., 2003; Huizinga et al., 2006). In contrast, the unitary nature of executive function is suggested by research indicating a single latent factor in early childhood (Wiebe et al., 2008, 2011; Hughes et al., 2010). In reconciling these discrepancies, it has been suggested that executive functions may in fact differ in structure across the preschool, primary, and adolescent years (Best and Miller, 2010). For instance, there is evidence supporting the unity of executive functions in the preschool years (Tsujimoto et al., 2007; Wiebe et al., 2008, 2011; Hughes et al., 2010), which fractionate in the primary school years (remaining as related, yet dissociable functions into adulthood; Miyake et al., 2000; Lehto et al., 2003; Huizinga et al., 2006). Recent longitudinal factor analytic evidence provides further support for this developmental differentiation of executive functions (Brydges et al., 2014).

Yet conflicting results continue to suggest the presence of multiple executive functions, even in the early years. For instance, Miller et al. (2012) found a two-factor structure (i.e., WM and inhibition) of executive functions among preschoolers. The longitudinal results of Usai et al. (2014) also supported two executive function factors among 5- and 6-year old children, although they characterize these as WM and shifting. Given continued inconsistency in findings, it has been suggested that these discrepancies may be a product of: (i) the common practice of collapsing participants into overly large age bands, which may obscure rapid changes in the quality and structure of executive functions with increasing age (e.g., Hughes, 1998; Klenberg et al., 2001; Lehto et al., 2003; Wiebe et al., 2008; Miller et al., 2012); and (ii) problematic selection of tasks and indices, which may preclude precision in measurement of young children’s executive functioning (Miller et al., 2012).

The current study thus sought to evaluate the proposed developmental differentiation model of executive functions (Tsujimoto et al., 2007; Brydges et al., 2014) in the preschool years, using well-established measures and narrower age intervals than have previous studies (i.e., 6 months). Specifically, cross-sectional analyses within each age band were used to examine relationships between executive functions (i.e., WM, inhibition, and shifting). Given the model’s hypothesis of undifferentiated executive functioning among preschoolers, yet the developmental differentiation of executive functions with increasing age, it was expected that executive functions would remain highly related in each age group but would display gradual differentiation as age increased.

## Materials and Methods

### Participants

Participants were 281 children aged 3–4 years (*M* = 4.11, SD = 0.59). Participants were recruited from 11 Australian preschool centers managed by a not-for-profit organization. Data from one participant was excluded due to early withdrawal resulting in fewer than 50% of tasks being completed. The final sample consisted of 55 younger 3-year olds (range = 3.00–3.49 years;* M* = 3.28, SD = 0.15), 70 older 3-year olds (range = 3.50–3.99 years;* M* = 3.74, SD = 0.14), 78 younger 4-year olds (range = 4.00–4.49 years;* M* = 4.23, SD = 0.14), and 77 older 4-year olds (range = 4.50–4.99 years;* M* = 4.73, SD = 0.14). Fifty-two percent of participants were girls (*n* = 155), with a relatively even distribution of boys and girls within each age band. All children were native speakers of English. Parental consent, as well as the child’s verbal assent, was collected as a condition of participation.

### Measures

#### Backward Word Span

The Backward Word Span task (based on the protocols of Davis and Pratt, 1995), designed to measure the maximum number of items that concurrently can be activated and manipulated in WM, has been used extensively with preschoolers (Davis and Pratt, 1995; Carlson, 2005). Previous research has found good test-retest reliability with this task (Muller et al., 2012). This task requires participants to repeat a sequence of spoken words in reverse order. The task began with instructions and demonstration using a three-stimulus practice item combining visual and verbal presentation. This was followed by 3 two-stimulus, verbal-only practice trials. Successful completion of at least one practice trial resulted in the child progressing through tests lists of increasing length (i.e., two trials at each of two, three and four stimuli list lengths). The task was discontinued whenever a child was unable to correctly reverse at least one of the two trials within a given list length. Scores, which could range from 0 to 7, reflect the highest list length at which the child was able to complete at least one trial. If the child failed to correctly reverse a list length of two, they were assigned a score of one if they could correctly recite the two-stimulus item in unreversed order (Carlson, 2005).

#### Dimensional Change Card Sort (DCCS)

The Dimensional Change Card Sort (DCCS; based on protocols of Zelazo, 2006), a measure of shifting, has been used extensively with preschool-aged children (Zelazo, 2006; Miller et al., 2012), with research suggesting good test-retest reliability of this task (Beck et al., 2011). To start, the initial pre-switch condition required children to sort cards (i.e., red rabbits, blue boats) by a randomly selected sorting dimension (color or shape) into one of two boxes (identified by a blue rabbit or a red boat). After one demonstration trial and two practice trials, the tester then only reiterated the relevant sorting rule as they presented test trial cards. In the post-switch phase, children were required to sort cards by the other sorting dimension. The tester again began each trial by reiterating the relevant sorting rule and then presented a card for sorting. If the child correctly sorted at least five of the six post-switch cards, they proceeded to the border phase of the task. In this phase, children were required to sort by color if the card had a black border or sort by shape if the card had no black border. After a demonstration trial and two practice trials, the tester again reiterated the sorting rule prior to presenting a test card for sorting. For all conditions, cards were ordered such that a particular stimulus was never presented more than twice in a row. Scores, which could range from 0 to 12, represent the number of correct card sorts after the pre-switch phase.

#### Go/No-Go (GNG)

The go/no-go paradigm has been used extensively to measure inhibition in preschool-aged children (Miller et al., 2012; Wiebe et al., 2012), with previous research indicating acceptable test–retest reliability of GNG paradigms (Willoughby and Blair, 2011). The current variant (following the protocols of Wiebe et al., 2012) required participants to ‘catch fish’ by pressing a computer key and ‘avoid sharks’ by withholding this response. That the majority of stimuli were fish potentiated the ‘go’ response and required interruption of this response on ‘no-go’ trials. The task was introduced in the following sequence: instructions for fish, followed by five consecutive ‘go’ practice trials; instructions for sharks, followed by five consecutive ‘no-go’ practice trials; then a recap of instructions, followed by a mixed block of 10 practice trials (eight fish, two sharks). Feedback was provided for all practice trials. The task proceeded in three mixed blocks of 25 stimuli, each consisting of 80% go trials. A block design was used to provide participants with a short break before having to refocus their attention. Stimuli were presented in random order for 1500 ms each, followed by a 1000 ms interval between stimuli. Between each test block, the tester reiterated rules for responding. Scores, which could range from 0 to 100%, represent proportional accuracy on no-go trials.

### Procedure

All children were administered this battery of executive function tasks in the following order: WM (Backward Word Span), shifting (DCCS), and inhibition (GNG). To optimize children’s attention and engagement, all tasks were administered individually in a single 30-minute testing session, in a quiet area of the child’s preschool. When necessary, short breaks were used to maintain children’s interest and attention.

## Results

Initial data screening indicated that some participants displayed problematic patterns of GNG responding. Individual blocks were removed for cases of indiscriminant responding (go trials: >80% accuracy, no-go trials <20% accuracy) or non-responsiveness (go trials: <20% accuracy, no-go trials: >80% accuracy). Data was also removed if the child was unable to understand task requirements (e.g., when instructed to say words backward, they responded ‘backward’). This resulted in 6.2% of data being unavailable (due to early withdrawal) or removed, the distribution of which was fairly consistent across the age groups (ranging from 3.4% for the younger 4-year olds to 8.4% for the younger 3-year olds). After this screening process, no extreme observations were noted (defined as greater than 3 SDs from the mean). All unavailable and removed data was treated as missing data in subsequent analyses.

**FIGURE 1:**
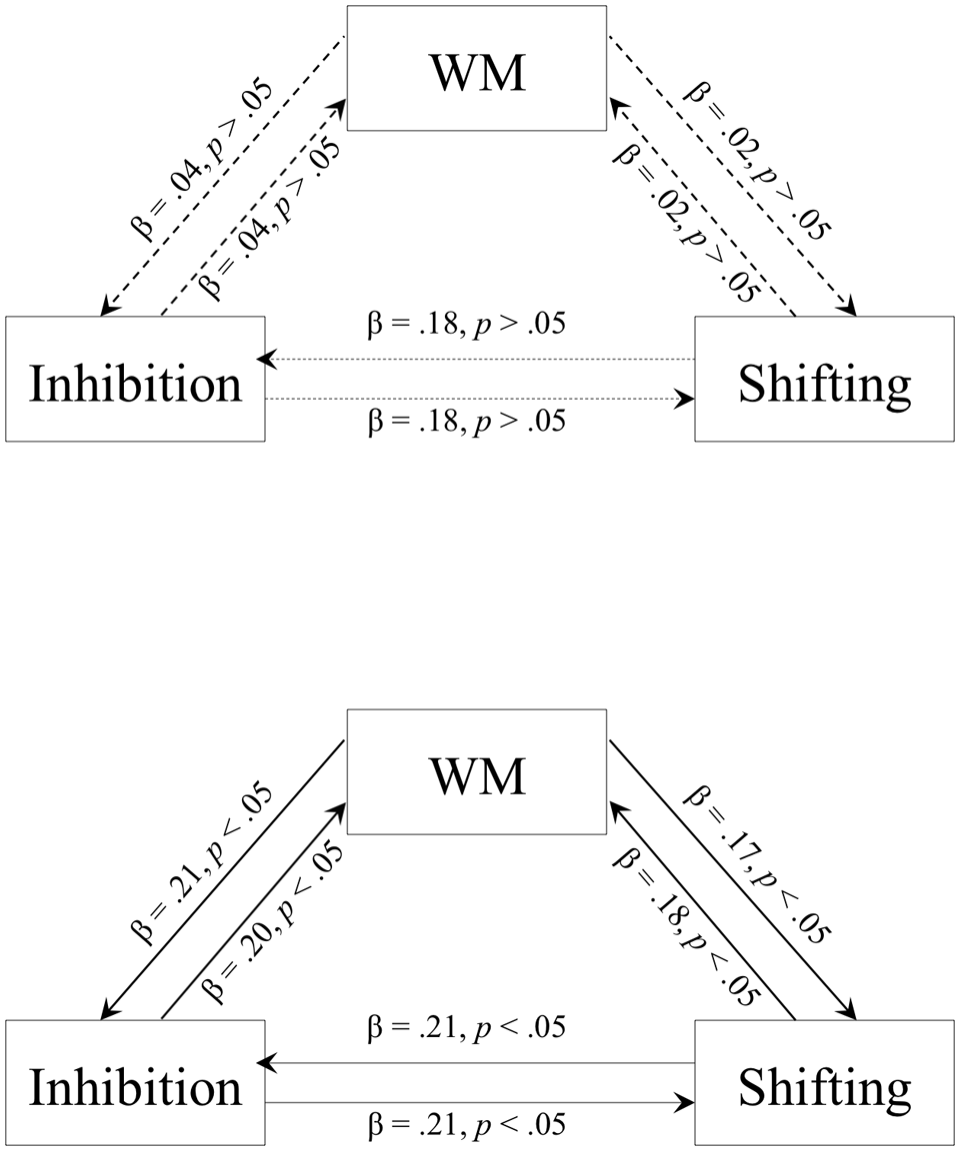
**Path diagrams displaying the relationship between three executive function tasks for 3-year olds (*n* = 99; **Top**) and 4-year olds (*n* boldsymbol = 136; **Bottom**) based on multiple, two-step hierarchical multiple regression analyses to control for the effect of age.** Each path is depicted with a standardized partial regression coefficient (β) and *p*-value associated for that specific path, controlling for age. Working memory (WM) was indexed by a Backward Word Span task. Shifting was indexed by a Dimensional Change Card Sorting (DCCS) task. Inhibition was indexed by a go/no-go (GNG) task.

To evaluate the hypothesis of the undifferentiated executive functioning of preschool-aged children, two-step hierarchical multiple regression analyses were performed, regressing age and scores for two executive function measures on scores for the third executive function measure (for similar analyses in the early primary school years, see Tsujimoto et al., 2007). To maximize statistical power, these analyses were conducted separately for 3-year olds and 4-year olds (although see **Table 1** for inter-task correlations for 6-month age groups). Altogether, three multiple regression analyses were run for each age group, each of which used a different executive function measure as the dependent variable (see **Figure 1** for presentation of these results as path diagrams). Contrary to our hypotheses, results indicated that performance on each executive function task was significantly predicted by performance on all other tasks for the 4-year old group (standardized βs ranged from 0.17 to 0.21), yet there were no significant paths in the 3-year old group (standardized βs ranged from 0.02 to 0.18). In order to statistically compare the regression coefficients between the age groups, subsequent analyses re-ran these regression analyses for the full sample, adding an interaction term (the product of group and executive function task score) to evaluate the null hypothesis that regression weights of the 3- and 4-year old groups were equivalent. Results indicated significance of the interaction term when regressing inhibition and shifting on WM (*p*s < 0.05), but non-significance when regressing shifting on inhibition (and vice versa). This indicates a significant difference between the age groups for all but the shifting-inhibition paths.

**Table 1:** Correlations between tasks as a function of age group.

	Younger 3	Older 3	Younger 4	Older 4
BWS-DCCS	0.13	-0.08	0.14	0.28^∗^
BWS-GNG	-0.01	0.06	0.22	0.29^∗^
DCCS-GNG	0.13	0.19	0.24^∗^	0.24^∗^

Values are inter-task correlation coefficients as a function of age group. BWS, Backward Word Span; DCCS, Dimensional Change Card Sort; GNG, go/no-go; ^∗^*p* < 0.05.

**FIGURE 2:**
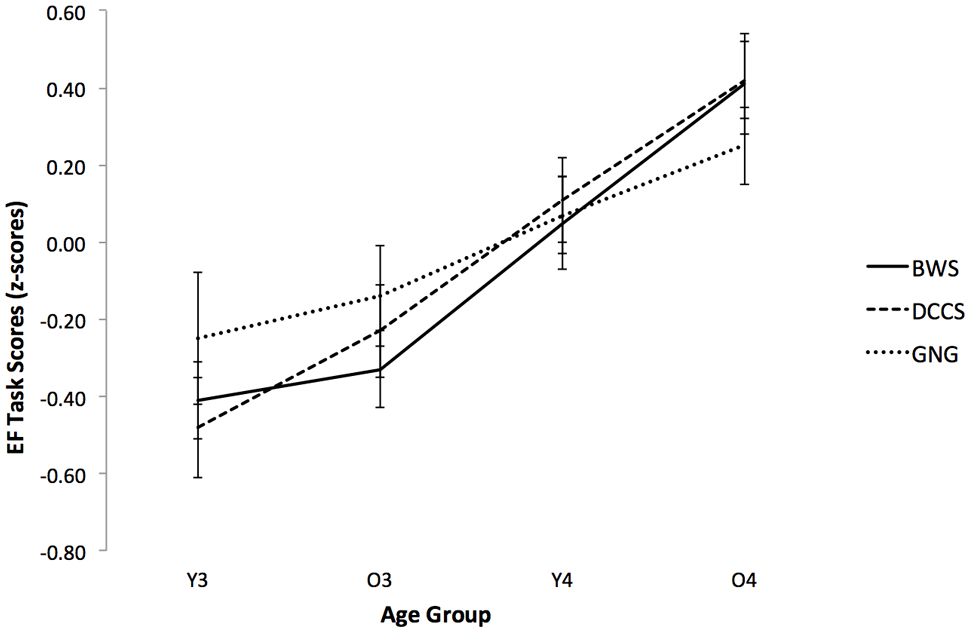
**Standardized performance (*z*-scores) for each executive function task as a function of age group.** BWS, Backward Word Span; DCCS, Dimensional Change Card Sort; GNG, Go/No-Go. Error bars represent ±1 SE.

To further examine the development of preschoolers’ executive functioning, analyses of variance compared the performance of participants separated into 6-month age bands (i.e., younger 3-year olds, older 3-year olds, younger 4-year olds, older 4-year olds). Descriptive statistics for these groups are provided in **Table 2**. ANOVAs yielded main effects of age for all tasks: Backward Word Span, *F*(3,250) = 7.62, *p* < 0.001, η^2^ = 0.11; DCCS, *F*(3,277) = 11.25, *p* < 0.001, η^2^ = 0.11; and GNG, *F*(3,265) = 3.24, *p* = 0.023, η^2^ = 0.04. *Post hoc* analyses indicated that for the Backward Word Span task, older 4-year olds outperformed all other age groups. For DCCS, older 4-year olds outperformed 3-year olds and younger 4-year olds outperformed younger 3-year olds. For GNG, older 4-year olds scored higher than younger 3-year olds (for presentation of these scores as standardized patterns of change, see **Figure 2**). Of note is the increased precision gained by analyzing differences across smaller age bands, compared to analyses using 1-year age bands (which indicated significant difference between 3- and 4-year olds, *p*s < 0.05).

**Table 2:** Descriptive statistics of executive function performance by age group.

	Younger 3	Older 3	Younger 4	Older 4	Overall	
Measure	M (SD)	M (SD)	M (SD)	M (SD)	M (SD)	Range
BWS	0.73 (0.62)	0.81 (0.72)	1.16 (0.97)	1.50 (1.05)	1.09 (0.93)	0–4
DCCS	3.06 (3.92)	4.11 (4.27)	5.56 (4.17)	6.88 (3.78)	5.07 (4.26)	0–12
GNG	0.62 (0.28)	0.65 (0.24)	0.70 (0.21)	0.74 (0.20)	0.68 (0.23)	0–100%

BWS, highest list length at which the child was able to complete at least one trial of the Backward Word Span task; DCCS, the number of correct card sorts after the pre-switch phase of the Dimensional Change Card Sort task; GNG, proportional accuracy on no-go trials of the go/no-go task.

## Discussion

Recent models and evidence of executive function development suggest that the early years of life are characterized by undifferentiated executive functioning, which fractionates into discrete yet related executive functions beginning in the primary school years (Tsujimoto et al., 2007; Brydges et al., 2014). This model of executive function development predicts highly related performance across WM, inhibition, and shifting tasks in the preschool years. It further suggests that the relationships between executive functions should gradually weaken with age into adulthood, coinciding with the fractionation of executive functions into related, dissociable functions. The current study sought to evaluate these predictions using well-established measures of executive functioning and more precise age groups than have previous studies. In contrast to this model of undifferentiated executive functioning becoming increasingly differentiated with age, however, current results suggest that young preschoolers’ executive function task performance was largely unrelated (evidenced by independence of executive components in multiple regression analyses). The executive functions of the older preschoolers, in contrast, were increasingly related (evidenced by increasing strength of inter-task correlations and multiple regression analyses indicating that all executive components were significantly related). This suggests that a single developmental trajectory toward increasing differentiation of executive functions may be insufficient to fully explain the early development of executive functioning.

Current results highlight the potential pitfalls of collapsing participants into overly large age groups when investigating the development of executive function, especially in the preschool years. This loss of precision in measurement may explain why some factor analytic studies of executive functions in the preschool years yield multiple distinct factors. For instance, Miller et al.’s (2012) collapsing of large age groups (i.e., 3–5 year olds), whose executive functions may have differed in structure according to current results, may have obscured an accurate picture of preschoolers’ executive function development. Although Wiebe et al. (2011) found similar evidence for a one-factor model of executive function using finer age groupings (i.e., 3-year olds), it is notable that their two-factor model actually provided marginally better fit to their data (a one-factor model was chosen on the basis of providing similarly good model fit, yet enhanced parsimony). These issues are further exacerbated by differences in the executive functions modeled, tasks selected, and the selection of performance indicators. For instance, McAuley and White’s (2011) finding of a stable organization of executive functions from early childhood – which contrasts evidence of the increasing fractionation of these functions – may have been influenced by the inclusion of a processing speed factor (a less conventional addition, with many studies instead including a ‘shifting’ factor) and the ages combined for their early childhood (6–8 year olds), late childhood (9–12 year olds), adolescence (13–17 year olds), and young adulthood samples (18–24 year olds).

Our ANOVA results provide further evidence advocating for adopting finer age groupings in developmental studies. That is, our finding that older 4-year olds tended to outperform the younger and older 3-year olds contrasts re-analysis of the data using 1-year age bands, which suggested that the 4-year old group outperformed the 3-year old group. Although there have already been efforts to elucidate the developmental trajectories of distinct executive functions (Best and Miller, 2010), and even executive function measures (Carlson, 2005), our results suggest that these efforts would benefit from adopting similarly fine age groups (in order to consider not only quantitative increases in performance, but also potential qualitative changes in the structure of executive functions). This is likely especially important in the early years, given the rapid development of executive functions occurring in this period. A precise picture of the developmental trajectories of the different executive functions is important not only for advances in theory, but also for identifying ages at which individual executive functions are most susceptible to intervention (whether positive or negative). For instance, it may be that function-specific strategies (e.g., chunking the contents of WM) are optimally effective only after fractionation in the primary years.

The current results thus contrast a model of initially undifferentiated executive functioning, which fractionates into discrete yet related executive functions. Specifically, current results suggest that preschoolers’ executive functioning may initially present as unrelated processes, yet undergo a period of integration in the preschool years. This suggestion is consistent with longitudinal evidence of increasing correlations between executive function tasks across the preschool years (Hughes, 1998). In fact, this period of integration may extend into the early primary years, as suggested by Tsujimoto et al. (2007) findings that executive functions were more strongly related in early-primary school students (5–6 years of age) than in later-primary school students (8–9 years of age). It thus may be the case that, after early integration in the preschool and early primary years, executive functions begin to fractionate in the later primary school years. If so, this would explain why many factor analytic studies of children’s executive functioning in the late preschool and early primary years yield a single executive function factor (Hughes et al., 2010; Welsh et al., 2010; Wiebe et al., 2011), whereas multiple executive functions are typically extracted in factor analytic studies with older children and adults (Miyake et al., 2000; Klenberg et al., 2001; Lehto et al., 2003; Huizinga et al., 2006). In fact, Garon et al.’s (2008) synthesis of executive function studies in the preschool years led them to conclude, “before 3 years of age, basic skills needed for component [executive functions] emerge, whereas development after age 3 appears to be an integrative period in which basic skills become coordinated” (p. 53). However, whether this period of early integration is related to variation in executive functions (increasing integration of executive function processes) or contributing non-executive abilities (e.g., problem solving strategies) is an area requiring further investigation.

Although the current study provides unique evidence toward reconciling existing debates in the executive function literature, interpretation of these results must be considered in light of the cross-sectional nature of the data and the limited number of tasks used to collect these data. That is, although the strongest analyses appropriate for our data were applied, the cross-sectional nature of the data did not allow for hierarchical linear modeling, which would have permitted statistical comparison of the developmental trajectories of each executive function. Nevertheless, standardized cross-sectional trends in the current data were relatively consistent across the three executive functions (as would be expected if these functions are becoming increasingly integrated). A further limitation of this study was the adoption of only a single indicator of each function, which conflates ability-specific and task-specific variance (a task impurity issue) and precludes latent variable analyses. For instance, strengthening inter-task correlations may be a product of maturation or performance strategies (among other possible explanations). In the current study, however, that inter-task correlations were largely maintained after controlling for age suggests that these results cannot be solely ascribed to general cognitive maturation. Although possibly divergent performance strategies cannot be similarly accounted for in the current data, it has been suggested that acquisition and deployment of different strategies mirrors developmental change in underlying neural systems (Tsujimoto et al., 2007). Even differential strategy adoption may thus signal the integration or fractionation of executive functions. Nevertheless, longitudinal research that adopts multiple indices of each executive function is required to further substantiate this initial evidence of early executive function development.

## Conclusion

The current data provide evidence to contrast the model of initially undifferentiated executive functioning, which fractionates into discrete yet related executive functions. The current data provide evidence that the preschool years may be a period of integration of initially unrelated executive processes, rather than a unified executive resource in the early preschool years. Our findings also highlight the need to consider the potentially changing nature and structure of young children’s executive functioning, prior to collapsing preschool-aged children into a single ‘homogeneous’ group. Such methods, while common, may serve to obscure genuine developmental trends and further complicate, rather than clarify, issues under investigation.

## Conflict of Interest Statement

The authors declare that the research was conducted in the absence of any commercial or financial relationships that could be construed as a potential conflict of interest.
